# The Genus *Leccinum* (Boletaceae, Boletales) from China Based on Morphological and Molecular Data

**DOI:** 10.3390/jof7090732

**Published:** 2021-09-06

**Authors:** Xin Meng, Geng-Shen Wang, Gang Wu, Pan-Meng Wang, Zhu L. Yang, Yan-Chun Li

**Affiliations:** 1 Key Laboratory for Plant Diversity and Biogeography of East Asia, Kunming Institute of Botany, Chinese Academy of Sciences, Kunming 650201, China; Mengxin@mail.kib.ac.cn (X.M.); wanggengshen@mail.kib.ac.cn (G.-S.W.); wugang@mail.kib.ac.cn (G.W.); wangpanmeng@mail.kib.ac.cn (P.-M.W.); 2 Yunnan Key Laboratory for Fungal Diversity and Green Development, Kunming Institute of Botany, Chinese Academy of Sciences, Kunming 650201, China; 3 College of Life Sciences, University of Chinese Academy of Sciences, Beijing 100049, China

**Keywords:** boletes, taxonomy, morphology, phylogeny, new taxa

## Abstract

*Leccinum* is one of the most important groups of boletes. Most species in this genus are ectomycorrhizal symbionts of various plants, and some of them are well-known edible mushrooms, making it an exceptionally important group ecologically and economically. The scientific problems related to this genus include that the identification of species in this genus from China need to be verified, especially those referring to European or North American species, and knowledge of the phylogeny and diversity of the species from China is limited. In this study, we conducted multi-locus (nrLSU, *tef1-α*, *rpb2*) and single-locus (ITS) phylogenetic investigations and morphological observisions of *Leccinum* from China, Europe and North America. Nine *Leccinum* species from China, including three new species, namely *L. album*, *L.* *parascabrum* and *L.* *pseudoborneense*, were revealed and described. *Leccinum album* is morphologically characterized by the white basidioma, the white hymenophore staining indistinct greenish blue when injured, and the white context not changing color in pileus but staining distinct greenish blue in the base of the stipe when injured. *Leccinum* *parascabrum* is characterized by the initially reddish brown to chestnut-brown and then pale brownish to brown pileus, the white to pallid and then light brown hymenophore lacking color change when injured, and the white context lacking color change in pileus but staining greenish blue in the base of the stipe when injured. *Leccinum* *pseudoborneense* is characterized by the pale brown to dark brown pileus, the initially white and then brown hymenophore lacking color change when injured, and the white context in pileus and stipe lacking color change in pileus but staining blue in stipe when bruised. Color photos of fresh basidiomata, line drawings of microscopic features and detailed descriptions of the new species are presented.

## 1. Introduction

The genus *Leccinum* Gray is a species-rich genus of Boletaceae and is characterized by a whitish or yellow hymenophore, a white to cream context unchanging or staining blue or red when injured, a brown to blackish scabrous to dotted squamules on the surface of the stipe, and comparatively long and smooth basidiospores. Generally, most species of the genus are widely spread in the subarctic, boreal, temperate and Mediterranean regions, with a few secondary expansions to the neotropics [1,2,3,4,5,6,7,8,9,10,11,12]. Species in *Leccinum* are both ecologically and economically important. Most species of this genus exhibit mycorrhizal host specificity. Species of *Leccinum* sect. *Scabra* Smith & Thiers are associated with plants of *Betula*, while species of *L.* sect. *Fumosa* (A.H. Smith, Thiers & Watling) Gelardi are associated with plants of *Populus*. In *L.* sect. *Leccinum*, species are found exclusively associated with plants of *Populus* (e.g., *L. albostipitatum* den Bakker & Noordel. and *L. insigne* A.H. Sm., Thiers & Watling), *Betula* (e.g., *L. atrostipitatum* A.H. Sm., Thiers & Watling), Pinaceae (e.g., *L. vulpinum* Watling and *L. piceinum* Pilát & Dermek) and Ericaceae that form arbutoidmycorrhizas (e.g., *L. manzanitae* Thiers and *L. monticola* Halling & G.M. Muell.). However, there are species in section *Leccinum* that are not host specific, i.e., *L. aurantiacum* (Bull.) Gray. This species is associated with plants of *Betula*, *Populus*, *Quercus*, *Salix* and sometimes with *Tilia* [13,14]. Some species of this genus are well-known edible mushrooms, such as *L. quercinum* (Pilát) E.E. Green & Watling, *L. scabrum* (Bull.) Gray and *L. versipelle* (Fr. & Hök) Snell, which are collected in China during the mushroom season.

The genus *Leccinum* was established by Gray in 1821 [13], based on the type species *L. aurantiacum*. Subsequently, more and more mycologists noticed the morphological distinctness and described many new species of this genus. As currently circumscribed, the genus comprises roughly 150 species [1,2,3,6,7,8,9,10,11,12,13,14,15,16,17,18,19,20,21,22,23,24,25,26,27,28,29,30,31,32,33,34,35,36,37,38,39,40,41,42,43,44,45,46,47,48,49,50,51,52,53,54,55,56]. North America is the species diversity center of this genus, and in total 118 species have been recorded from this area [19]. Some of the most important works are the serial works of Smith and Thiers [1,15,16,17], in which three sections of this genus were proposed (*L.* sect. *Leccinum* Smith & Thiers, *L.* sect. *Luteoscabra* Smith & Thiers and *L.* sect. *Scabra*), with 68 species described from Michigan. Twelve species from Central America were described: one species from Belize, eight species from Costa Rica and three species from Colombia [20,21,22,23,24]. In Europe, Singer divided species of this genus into four sections, including two known sections, *L.* sect. *Luteoscabra* and *L.* sect. *Leccinum*, and two newly proposed sections, *L.* sect. *Roseoscabra* and *L.* sect. *Eximia* [3]. In Singer’s infrageneric classification, *L.* sect. *Scabra*, established by Smith and Thiers, was merged to *L.* sect. *Leccinum*. Recent molecular phylogenetic evidence has revealed that species of *L.* sect. *Luteoscabra*, *L.* sect. *Roseoscabra* and *L.* sect. *Eximia* belong to divergent clades of Boletaceae and represent many new genera (32,52–54). Thus, the genus *Leccinum* is restricted to the section *Leccinum* (Singer’s infrageneric classification) [3]. den Bakker and Noordelos revised the European *Leccinum* species based on morphology and nrLSU sequences and documented sixteen species [14]. In their subsequent study, they treated the three subclades revealed by den Bakker et al. in *L.* section *Leccinum* [33,57] as three subsections (viz. *L.* subsect. *Leccinum*, *L.* subsect. *Fumosa* A.H. Sm., Thiers & Watling and *L.* subsect. *Scabra* Pilat & Dermek) [14]. This infrageneric subdivision was followed in the treatment of the genus in this study. In the Southern Hemisphere, four species have been reported, including one from New Zealand and three from Australia [27,28,29].

In Asia, six species of *Leccinum* have been reported from Malaysia [6]; ten species from Japan [7,8,9,10]; and a total of 31 species have been reported from China based on an extensive literature review [34,35,36,38,39,40,41,42,43,44,45,46,47,48,49,50,51,52,56]. Among these Chinese species, twelve species, viz. *L. albellum* (Peck) Singer, *L. chromapes* (Frost) Singer, *L. crocipodium* (Letell.) Watling, *L. eximium* (Peck) Singer, *L. extremiorientale* (Lar. N. Vassiljeva) Singer, *L. griseum* (Quél.) Singer, *L. hortonii* (A.H. Sm. & Thiers) Hongo & Nagas., *L. nigrescens* (Richon & Roze) Singer, *L. rubropunctum* (Peck) Singer, *L. rubrum M. Zang*, *L. rugosiceps* (Peck) Singer and *L. subglabripes* (Peck) Singer have been transferred to other genera [5,11,35,52,53,54,55]; eight species, viz. *L. duriusculum* (Schulzer ex Fr.) Singer, *L. intusrubens* (Corner) Høil., *L. oxydabile* (Singer) Singer, *L. quercinum*, *L. rufum* (Schaeff.) Kreisel, *L. subleucophaeum* E.A. Dick & Snell, *L. subradicatum* Hongo and *L. variicolor* Watling were reported without specimen support [39,40,41,42,43,49,51]; and eleven species, viz. *L. ambiguum* A.H. Sm. & Thiers, *L. atrostipitatum* A.H. Sm., Thiers & Watling, *L. aurantiacum*, *L. holopus* (Rostk.) Watling, *L. olivaceopallidum* A.H. Sm., Thiers & Watling, *L. potteri* A.H. Sm., Thiers & Watling, *L. roseofractum* Watling, *L. scabrum*, *L. subgranulosum* A.H. Sm. & Thiers, *L. subleucophaeum* var. *minimum* C.S. Bi and *L. versipelle* were reported with specimen citations [34,38,44,45,46,47,48]. Among these eleven species reported with specimen citations, only *L. subleucophaeum* var. *minimum* was originally described from China, and the remaining species were identified as species originally described from Europe and North America based on general morphological similarities. Indeed, a few species described from Europe and North America do occur in China, especially in northeastern and northwestern China. However, most species found in China have evolved independently in the southern part of China. Thus, identification of the Chinese *Leccinum* species needs to be reconfirmed.

In this study, we used both morphological data and molecular sequences from the nuclear ribosomal internal transcribed spacer (ITS), the large subunit of the nuclear ribosomal RNA (nrLSU), the translation elongation factor 1-alpha (*tef1-α*) and the RNA polymerase II second largest subunit (*rpb2*), together with ecological data to (1) elucidate species diversity of *Leccinum* in China; (2) evaluate the phylogenetic relationships of species within *Leccinum*; (3) make morphological and ecological comparisons between closely related species.

## 2. Materials and Methods

### 2.1. Taxon Sampling

Nineteen specimens of the genus *Leccinum* from China were examined. For each collection, a part of the basidioma was dried with silica gel for DNA extraction. The remaining materials were then air-dried at 45–50 °C using an electric food dehydrator. Specimens studied in this work were deposited in the Herbarium of the Kunming Institute of Botany, Chinese Academy of Sciences (KUN). Genera are abbreviated as follows: *L.* for *Leccinum*, *Le.* for *Leccinellum*, *O.* for *Octaviania*, *R.* for *Rossbeevera*, *Ru.* for *Rugiboletus*, *T.* for *Turmalinea*, Ca. for *Castanopsis*, *Li*. for *Lithocarpus*, *P.* for *Pinus* and *Q.* for *Quercus*.

### 2.2. Morphological Observation

The macroscopic descriptions are based on the detailed field notes and photographs of fresh basidiomata. Color codes of the form “4B2” indicate the plate, row, and color block from Kornerup and Wanscher [58]. For microscopic studies, the microscopic features of each part of the basidioma were observed under microscope (Leica DM2000, Leica Microsystems, Wetzlar, Germany), including basidiospores, basidia, cheilocystidia, pleurocystidia and pileipellis, using 5% KOH as a mounting medium to revive the dried materials. Microscopic studies follow Zhou et al. [59]. In the description of Basidiospores, the abbreviation n/m/p means n basidiospores measured from m basidomata of p collections in 5% KOH solution. The notation of the form (a) b–c (d) stands for the dimensions of the basidiospores; the range b–c contains a minimum of 90% of the measured values, a or d given in parentheses stands for extreme values. Q is used to mean “length/width ratio” of a basidiospore in a side view; Q_m_ means average Q of all basidiospores ± sample standard deviation. Measurements of basidospores, cystidia, basidia and terminal cells in pileipellis are presented as length × width. All microscopic structures were drawn freehand from rehydrated material under the microscope with 10× eyepiece and 100× objective (the total magnification is 1000×).

### 2.3. Molecular Procedures

Genomic DNA was extracted from silica gel dried materials or herbarium specimens using the CTAB (Cetyltrimethyl ammonium bromide) method [60]. Polymerase chain reactions (PCRs) were performed to amplify partial sequences of nrLSU, *tef1-α, rpb2* and ITS using the extracted DNA. The nrLSU region was amplified with primers LROR/LR5 and LROR/LR3 [61]; *tef1-α* was amplified with primer pair EF1-983F and EF1-1567R [62]; *rpb2* was amplified with primers bRPB2-6F and bRPB2-7.1R [63] and ITS was amplified with primer pair ITS1 and ITS4 [64]. Protocols for the polymerase chain reactions (PCRs) and sequencing followed those in Wu et al. [65] and the references therein.

### 2.4. Sequence Alignments and Phylogenetic Analyses

The newly generated sequences of each locus were blasted in GenBank, and the most closely related sequences (nucleotide identities >95%) were downloaded for further alignment. Sequences were aligned separately for each of the loci using MAFFT v7.130b with the E-INS-I strategy and manually optimized on BioEdit v7.0.9 [66,67]. Two datasets, the ITS dataset and the multi-locus (nrLSU + *tef1-α* + *rpb2*) dataset, were analyzed using RAxML and Bayesian methods, respectively. For the multi-locus dataset, single-gene analyses were conducted to assess incongruence among individual genes using the ML method (results not shown). Because no well-supported bootstrap value (BS > 70%) [55] conflict was detected among the topologies of the three genes, their sequences were then concatenated together for further multi-locus analyses.

For ML analyses, the multi-locus and ITS datasets were analyzed using RAxML (https://www.phylo.org/, accessed on 26 August 2021) under the model GTRGAMMA [68]. Statistical supports for the phylogenetic analyses were determined using nonparametric bootstrapping with 1000 replicates. For BI analyses, the parameter model was selected by the Akaike information criterion (AIC) as the best-fit likelihood model with Modeltest 3.7 (Free Software Foundation, Boston, MA, USA) [69]. The models employed for each of the four loci were GTR + I + G for ITS, nrLSU and *tef1-α*, and SYM + I + G for *rpb2*. Posterior probabilities (PP) were determined twice by running one cold and three heated chains in parallel mode, saving trees every 1000th generation. Other parameters were kept at their default settings. Runs were terminated once the average standard deviation of split frequencies went below 0.01 [70]. Chain convergence was determined using Tracer v1.5 (http://tree.bio.ed.ac.uk/software/tracer/, accessed on 26 August 2021) to confirm sufficiently large ESS values (>200). Subsequently, the sampled trees were summarized after omitting the first 25% of trees as burn-in using the ‘sump’ and ‘sumt’ commands implemented in MrBayes.

## 3. Results

### 3.1. Molecular Phylogenetic Analysis

**Figure 1:**
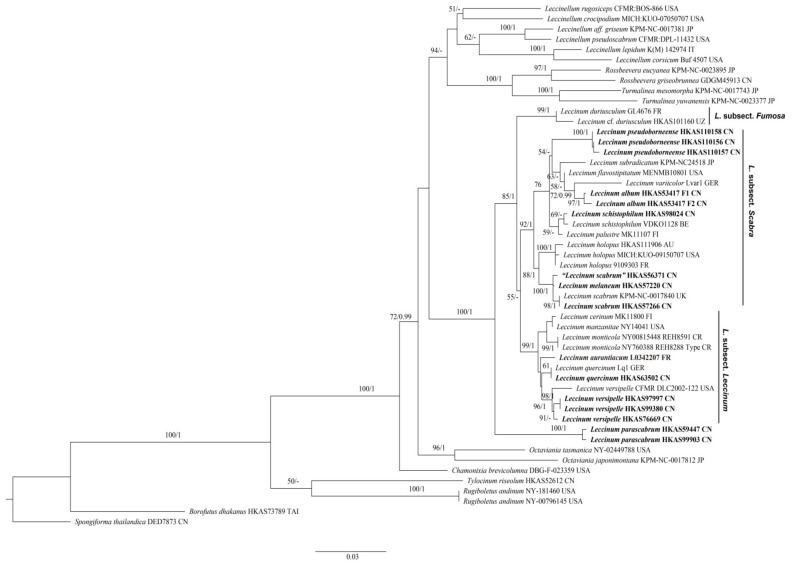
Maximum-likelihood phylogenetic tree generated from a three-locus (nrLSU + *tef1-α* + *rpb2*) dataset. BS > 50% in ML analysis and PP > 0.95 in Bayesian analysis are indicated as RAxML BS/PP above or below supported branches. Species of this genus from China and type species of this genus (*L. aurantiacum*) are indicated in bold. Voucher specimens and localities where the specimens were collected are provided behind the species names. AU = Austria, BE = Belgium, CN = China, CR = Costa Rica, FI = Finland, FR = France, GER = Germany, IT = Italy, JP = Japan, TAI = Thailand, UK =United Kingdom, USA = United States of America and UZ = Uzbekistan.

A total of 57 sequences, including fifteen for nrLSU, fifteen for *tef1-α*, fourteen for *rpb2* and thirteen for ITS, were newly generated in this study and aligned with sequences downloaded from GenBank. Sequences retrieved from GenBank and obtained in this study for the multi-locus phylogenetic analyses are listed in Table 1. The multi-locus dataset (Supplementary File S1) contained 122 sequences (49 for nrLSU, 41 for *tef1-α*, 32 for *rpb2*), representing 51 samples, and the alignment contained 2195 nucleotide sites, of which 530 were parsimony informative. *Borofutus dhakanus* Hosen & Zhu L. Yang and *Spongiforma thailandica* Desjardin, Manfr. Binder, Roekring & Flegel were chosen as the outgroup [71,72]. ML and Bayesian analyses produced very similar estimates of tree topologies, and thus only the tree inferred from ML analysis is displayed (Figure 1). The monophyly of *Leccinum* was highly supported (BS = 100% and PP = 1) in our analyses. Four main clades were recovered, and three of them correspond to the three known subsections, viz. *L.* subsect. *Leccinum*, *L.* subsect. *Fumosa* and *L.* subsect. *Scabra* of *L.* sect. *Leccinum* [14]. Three new species, namely *L. album*, *L. parascabrum* and *L. pseudoborneense*, were revealed in our multi-locus phylogenetic analyses. *Leccinum parascabrum* formed the remaining clade with BS = 100% and PP = 1, while *L. pseudoborneense* and *L. album* nested in *L.* subsect. *Scabra* and clustered together with *L. flavostipitatum* E.A. Dick & Snell, *L. subradicatum* and *L. variicolor* with low supported lineage (BS = 54%).

**Figure 2:**
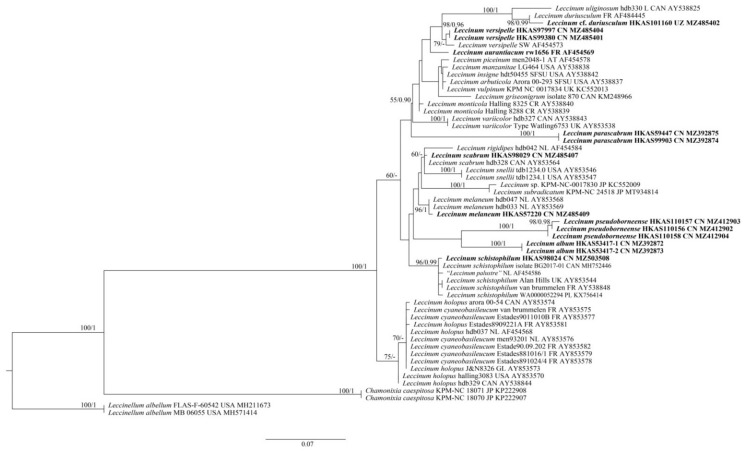
Maximum-likelihood phylogenetic tree generated from ITS dataset. BS > 50% in ML analysis is indicated above or below supported branches. Species of this genus from China and type species of this genus (*L. aurantiacum*) are indicated in bold. Voucher specimens, localities and GenBank numbers are provided behind the species names. AT = Austria, CAN = Canada, CN = China, CR = Costa Rica, FR = France, GL = Greenland, JP = Japan, NL = The Netherlands, PL = Poland, SW = Sweden, UK =United Kingdom, USA = United States of America and UZ = Uzbekistan.

For the ITS dataset, as revealed by den Bakker et al. [57] and our primary analysis, the ITS1 region contains a minisatellite, which is characterized by the repeated presence of CTATTGAAAAG and CTAATAGAAAG core sequences and mutational derivatives. Moreover, some species contain a minisatellite in the ITS2 region, e.g., the newly described species *L. album* (GenBank Acc. No.: MZ392872 for clone 1 and MZ392873 for clone 2), with a region of 212 bp that consists of tandem repeats (see Supplementary Material for details). Though there is length variation in either the ITS1 or ITS2 spacers, it can also provide some phylogenetic signals. We performed phylogenetic analyses of the ITS dataset. In this dataset (Supplementary File S2), 51 samples were included. The length of the dataset was 1416 bp, of which 377 were parsimony informative. *Leccinellum albellum* (Peck) Bresinsky & Manfr. Binder was chosen as outgroup. ML and Bayesian analyses also produced very similar estimates of tree topologies, and only the tree inferred from ML analysis is displayed (Figure 2). The monophyly of *Leccinum* was also well supported (BS = 100% and PP = 1) in our analyses. Three new species viz. *L. album*, *L. parascabrum* and *L. pseudoborneense*) were revealed. *Leccinum album* is closely related to *L. pseudoborneense* yet without statistical support, while *L. parascabrum* forms an independent lineage. Species to which *L. parascabrum* is phylogenetically related remain as yet unknown.

Our ML and Bayesian analyses of ITS and multi-locus datasets revealed the existence of eight *Leccinum* species from China, including five known species viz. *L. melaneum* (Smotl.) Pilát & Dermek, *L. quercinum*, *L. scabrum*, *L. schistophilum* and *L. versipelle* and three new species viz. *L. album*, *L. parascabrum*, and *L. pseudoborneense*. The final alignments of both datasets were deposited in TreeBASE (S27490).

### 3.2. Taxonomy

**Figure 3:**
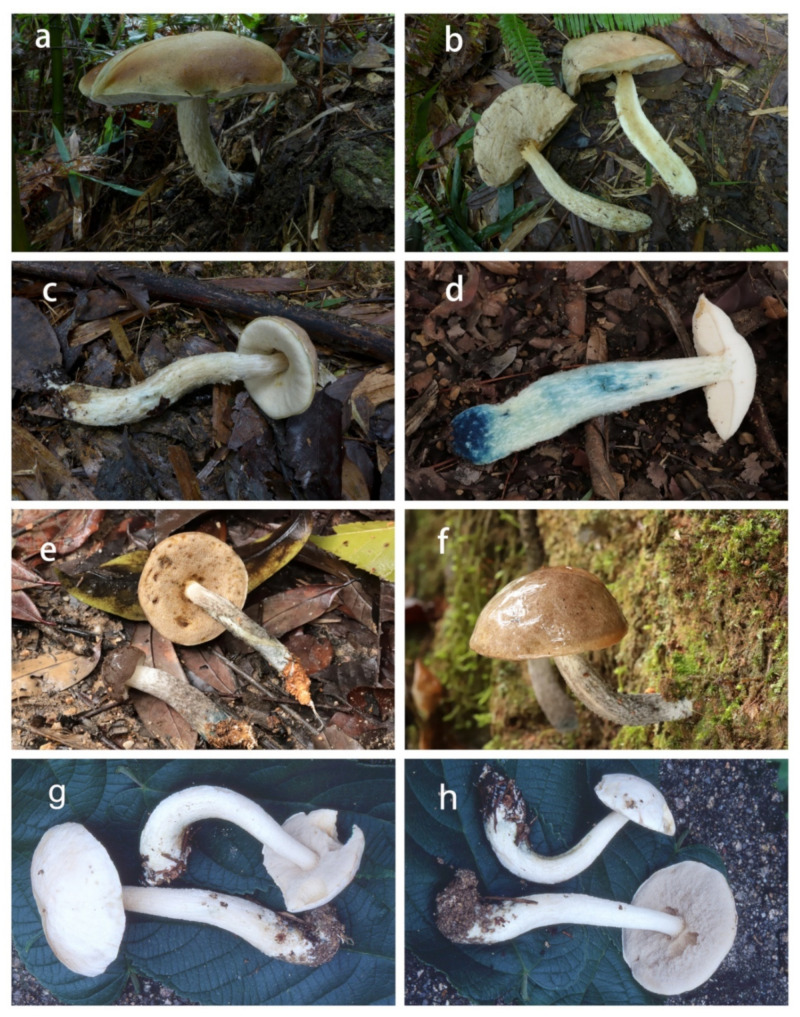
Basidiomata of *Leccinum* species. (**a**–**c**) *Leccinum parascabrum* (KUN-HKAS99903, holotype); (**d**–**f**) *Leccinum pseudoborneense* ((**d**) from KUN-HKAS110157; (**e**,**f**) from KUN-HKAS110156, holotype); (**g**,**h**) *Leccinum album* (KUN-HKAS53417, holotype).

**Figure 4:**
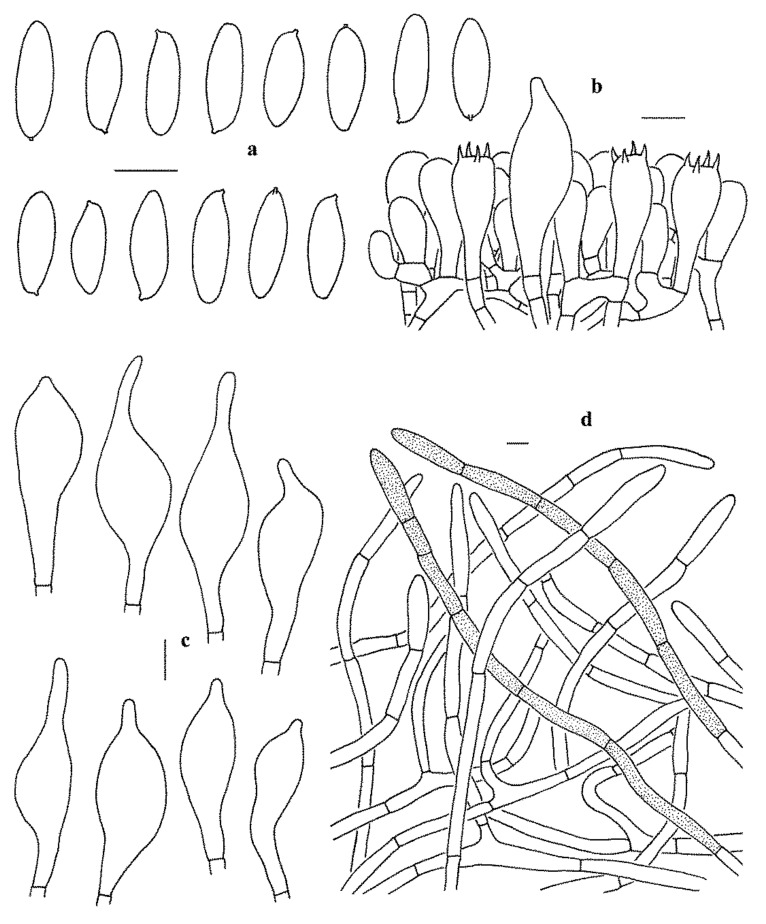
Microscopic features of *Leccinum album* (KUN-HKAS53417, holotype). (**a**) Basidiospores. (**b**) Basidia and pleurocystidium. (**c**) Cheilocystidia and pleurocystidia. (**d**) Pileipellis. Bars = 10 µm. Drawings by Y.-C. Li.

***Leccinum album*** X. Meng, Yan C. Li & Zhu L. Yang, sp. nov., (Figure 3g–h and Figure 4).

MycoBank: MB 838917.

*Diagnosis*: This species differs from other species in *Leccinum* in the combination of the entirely white pileus, the white pileal context not changing color when injured, the white hymenophore staining indistinct greenish blue when hurt, the white stipe coarsely covered with initially white and then darkened verrucose squamules, and the white stipe context always staining greenish blue at the base when injured.

*Holotype*: CHINA. Hunan Province: Chenzhou, Zhanghua County, Mangshan National Forest Park, E 112°92′, N 24°94′, alt. 850 m, associated with *Castanopsis fissa*, *Cyclobalanopsis glauca*, *Lithocarpus glabra* and *Pinus kwangtungensis*, 3 September 2007, Y.C. Li1072 (KUN-HKAS53417, GenBank Acc. No.: MZ392872 and MZ392873 for ITS, MW413907 and HQ326880 for nrLSU, MW439267 and HQ326861 for *tef1-α*, and MW439259 and MW439260 for *rpb2*).

*Etymology*: Latin “*album*” means white, referring to the color of the basidiomata.

Basidiomata small to medium-sized. Pileus 3–5.5 cm in diam., hemispherical when young, subhemispherical to convex or plano-convex when mature, white (1A1) when young, white to cream (2B2–3) when mature; surface covered with concolorous farinose to pubescent squamules; context 5–10 mm thick in the center of pileus, taste mild, white (1A1) to pallid, not changing color when bruised; Hymenophore adnate when young, adnate to slightly depressed around apex of stipe; surface white (1A1), staining indistinct greenish blue (25B5–7) when injured; pores subangular to roundish, 0.3–1.5 mm wide; tubes up to 5 mm long, white to dirty pinkish (13A2), not changing color when bruised. Stipe 8–10 × 0.8–1.2 cm, clavate to subcylindrical, always enlarged downwards; surface white (1A1), densely covered with white (1A1) verrucose squamules, staining light greenish blue at base when injured; context whitish (1A1), staining blue at base when injured; basal mycelium white (1A1), lacking color change when injured.

Basidiospores (40/2/1) 15–19 × 5–7 µm, Q = 2.5–3, Q_m_ = 2.75 ± 0.15, subfusiform to narrowly ellipsoid in side view with slight suprahilar depression, subcylindrical to fusiform in ventral view, smooth, somewhat slightly thick-walled (up to 0.5 μm thick), hyaline to yellowish in KOH, brownish yellow to olivaceous brown in Melzer’s Reagent. Basidia 23–33 × 10–13 µm, clavate, 4-spored, hyaline to yellowish in KOH, yellowish to brownish yellow in Melzer’s Reagent. Hymenophoral trama boletoid, hyphae subcylindrical, 4–10 µm wide, hyaline to yellowish in KOH, yellowish to yellow in Melzer’s Reagent. Cheilo- and pleurocystidia 42–60 × 11–17.5 µm, abundant, subfusiform to fusiform, thin-walled, yellowish in KOH, yellowish to brownish yellow in Melzer’s Reagent. Pileipellis a trichoderm, composed of more or less vertically arranged 5–10 µm wide hyphae, hyaline to yellowish in KOH, yellowish to yellow in Melzer’s Reagent. Pileal trama made up of 6–12 µm wide filamentous hyphae, thin-walled, yellowish in KOH, yellowish to brownish yellow in Melzer’s Reagent. Clamp connections absent in all tissues.

*Habitat and distribution*: Solitary or scattered in tropical forests dominated by plants of the families Fagaceae (*Castanopsis fissa*, *Cyclobalanopsis glauca* and *Lithocarpus glabra*) and Pinaceae (*Pinus kwangtungensis* or *P. armandii*); on acidic, humid and loamy soils; distribution insufficiently known, rather rare in China and currently found in central and southeastern China (Hunan and Fujian Provinces).

*Additional Specimen examined*: CHINA. Fujian Province: Jianning County, E 116°84′, N 26°83′, alt. 900 m, associated with *Castanopsis fissa*, *Cyclobalanopsis glauca* and *Pinus armandii*, 16 July 1971, N.L. Huang 716 (KUN-HKAS39522).

*Commentary*: *Leccinum album* is characterized by the white pileus, the white hymenophore staining indistinct greenish blue when hurt, the white stipe densely covered with initially white and then darkened scabrous squamules, the white context in pileus not changing color when injured, and the white context in stipe unchanging or only staining distinct greenish blue at base when injured. Morphologically, *L. album* is close to *L. holopus*, *L. cyaneobasileucum* Lannoy & Estadès and *Le. albellum* (Peck) Bresinsky & Manfr. Binder in similar pileus colors. However, *L. holopus*, originally described from Europe (Germany), differs from *L. album* in its medium to large basidiomata (pileus 4–10 cm wide), becoming more viscid pileus with age, pure white or dirty white to pale buff or pale pallid pileus always with a glaucous green tinge, long hymenophoral tubes measuring 9–15 mm long, narrow and subcylindrical hymenial cystidia measuring 30–50 × 7.5–12.5 µm, narrow pileipellis hyphae measuring 3.5–5 µm wide, and association with trees of the genus *Betula* (Betulaceae) [80,81,82]. *Leccinum cyaneobasileucum*, originally described from France, is different from *L. album* in its white or greyish brown to light brown pielus, woolly stipe surface, slender basidiospores with Q_m_ ≥ 3, relatively narrow hymenial cystidia measuring 32–44 × 5.5–7.5 µm, narrow pileipellis hyphae measuring 2–6.5 µm wide, and association with trees of the genus *Betula* [83]. *Leccinellum albellum*, originally described from New York, is characterized by its basidiomata not changing color when bruised and narrow basidiospores measuring 13–20 × 4–6 µm [16,17,30].

Phylogenetically, *L. album* is related to *L. variicolor* and *L. pseudoborneense* in the analyses of the multi-locus and ITS datasets, respectively (Figure 1 and Figure 2). However, *L. variicolor* differs from *L. album* in its white to grey or cream pileal context staining vinaceous to brown when bruised, white stipe context staining pink to coral red in the upper part and green-blue in the lower part when bruised and association with plants of *Betula* [81]. *Leccinum pseudoborneense* is different from *L. album* in its pale brown to dark brown pileus, white context in pileus and stipe staining blue when bruised, narrow basidiospores measuring (11) 12–19 (20) × 4–5 (6) µm, narrow hymenial cystidia measuring 28–40 × 4–10 µm, and distribution in southwestern China.

**Figure 5:**
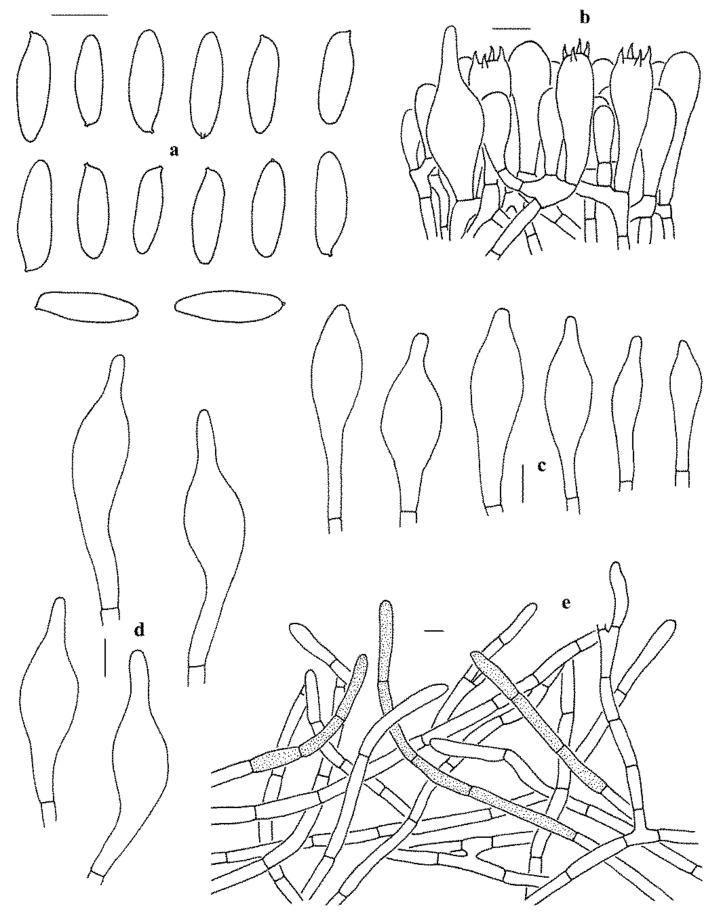
Microscopic features of *Leccinum parascabrum* (KUN-HKAS99903, holotype). (**a**) Basidiospores. (**b**) Basidia and pleurocystidium. (**c**) Cheilocystidia. (**d**) Pleurocystidia. (**e**) Pileipellis. Bars = 10 µm. Drawings by Y.-C. Li.

***Leccinum parascabrum*** X. Meng & Yan C. Li & Zhu L. Yang, sp. nov., (Figure 3a–c and Figure 5).

MycoBank: MB 838916.

*Diagnosis*: This species differs from other species in *Leccinum* by its initially reddish brown to chestnut-brown and then brown to pale brownish or even dirty white pileus, white pileal context lacking color change when injured, white to pallid and then light brown hymenophore lacking color change when injured, and the white stipe context staining greenish blue at the base when injured.

*Holotype*: CHINA. Hunan Province: Chenzhou, Zhanghua County, Mangshan National Forest Park, E 112°92′, N 24°94′, alt. 1100 m, associated with *Castanopsis fissa*, *Lithocarpus glabra* and *Pinus kwangtudgensis*, 12 September 2016, G. Wu 1784 (KUN-HKAS99903, GenBank Acc. No.: MZ392874 for ITS, MW413911 for nrLSU, MW439271 for *tef1-α*, and MW439264 for *rpb2*).

*Etymology*: Latin “*parascabrum*” refers to its similarity to *L. scabrum*.

Basidiomata small to medium-sized. Pileus 2.5–12.5 cm in diam., hemispherical when young, subhemispherical to convex or applanate when mature, reddish brown (12E8) to chestnut-brown (8C7–8) when young, brown (6C6) to pale brownish (7D7–8) or even dirty white (6A2) when mature; surface tomentose; context 6–13 mm thick in the center, white (1A1), not changing color when bruised; Hymenophore adnate when young, adnate to slightly depressed around apex of stipe; surface white to pallid (1A1) when young, and becoming light brown (6B4) when mature, not changing color when injured; tubes 6–14 mm long, 0.5–1.5 mm wide, creamy white (1A1), not changing color when bruised. Stipe 12–14 × 1.1–2.2 cm, clavate, swollen downwards, always staining greenish blue at base when injured; surface white (1A1), covered with initially white (1A1) to light beige (5A4) and then brownish (7D8) squamules; context white (1A1), staining greenish blue (25B6–7) at base when injured; basal mycelium white (1A1).

Basidiospores (80/2/2) 16–20 (–21) × 5–6 µm, Q = 3.2–3.8, Q_m_ = 3.43 ± 0.18, subfusiform to fusiform, slightly thick-walled (up to 0.5 μm thick), yellowish to yellowish brown in KOH, yellow to yellow-brown in Melzer’s Reagent. Basidia 24–33 × 8–12 µm, clavate, 4-spored, hyaline to yellowish in KOH, yellowish to yellow in Melzer’s Reagent. Hymenophoral trama boletoid, hyphae cylindrical, 3–7 µm wide, hyaline to yellowish in KOH, yellowish to yellow in Melzer’s Reagent. Cheilo- and pleurocystidia 34–68 × 7.5–16 µm, abundant, subfusiform to fusiform, thin-walled, yellowish to pale yellowish brown in KOH, yellowish brown to brown in Melzer’s Reagent. Pileipellis a trichoderm, composed of 5–9 µm wide filamentous hyphae, yellowish to pale brownish in KOH. Pileal trama made up of 5–10 µm wide filamentous hyphae, thin-walled, hyaline to yellowish in KOH, yellowish to brownish yellow in Melzer’s Reagent. Clamp connections absent in all tissues.

*Habitat and distribution*: Solitary or scattered in tropical forests dominated by plants of the families Fagaceae (*Lithocarpus glabra, Castanopsis fissa* and Ca. *hystrix*) and Pinaceae (*Pinus kwangtudgensis* or *P. yunnanensis*.); on acidic or slightly alkaline, loamy soils; distribution insufficiently known, rather rare in China, currently known from central and southwestern China (Hunan and Yunnan Provinces).

*Additional Specimen examined*: CHINA. Yunnan Province: on the way from Tengchong County to Longling County, E 98°59′, N 24°81′, alt. 2010 m, associated with *Lithocarpus glabra*, *Castanopsis hystrix* and *Pinus yunnanensis*, 19 July 2009, Y.C. Li 1700 (KUN-HKAS59447, GenBank Acc. No.: MZ392875 for ITS, MW413912 for nrLSU, MW439272 for *tef1-α*, and MW439265 for *rpb2*).

*Commentary: Leccinum parascabrum* is characterized by the initially reddish brown to chestnut-brown and later brown to pale brownish or even dirty white pileus, the white pileal context not changing color when injured, the white to pallid and then light brown hymenophore not changing color when injured, the white stipe context with greenish blue color change at the base when injured, and the relatively large basidiospores measuring 16–20 (–21) × 5–6 µm, Q = 3.2–3.8. *Leccinum parascabrum* generally shares the similar colors of pileus and hymenophore, and the similar slender stems with *L. duriusculum*, *L. griseonigrum* A.H. Sm., Thiers & Watling, *L. scabrum*, *L. uliginosum* A.H. Sm. & Thiers and *Le. pseudoscabrum* (Kallenb.) Mikšík. However, *L. duriusculum*, originally described from Europe, can be distinguished from *L. parascabrum* by its pale grey-brown to dark greyish or reddish brown pileus, white context staining violaceous pink when bruised but yellow-green to blue-green in the base of stipe, relatively small basidiospores measuring 11.5–15.5 × 4.5–6 µm [84]. *Leccinum griseonigrum*, originally described from North America, differs from *L. parascabrum* in its avellaneous to dingy cinnamon-buff pileus, white pileal context staining blue when bruised, relatively small basidiospores measuring 13–16 × 4–5.5 µm, and association with trees of the genus *Populus* [16]. *Leccinum scabrum* differs from *L. parascabrum* in its wrinkled pileus, pale white hymenophore, pinkish discoloration when injured, and never bluish color change at the base of stipe [13,14,81]. *Leccinum* *uliginosum*, originally described from North America, is different from *L. parascabrum* in its dark fuscous to drab-grey pileus, white context in pileus becoming reddish and then fuscous when bruised, relatively small basidiospores measuring 14–18 × 3.5–5 µm, and small and inconspicuous hymenial cystidia [17]. *Leccinellum pseudoscabrum* differs from *L. parascabrum* in its initially red to purplish brown and then blackish brown context color change when injured, and the palisadoderm pileipellis composed of subglobose cells [14]. *Leccinum parascabrum* also shares the similar colors of pileus and hymenophore and the bluish color change at the base of stipe with *L. variicolor*. However, *L. variicolor* is different from *L. parascabrum* in its white to grey or cream pileal context staining vinaceous to brown when bruised, white stipe context staining pink to coral red in the upper part and green-blue in the lower part when bruised, relatively small basidiospores measuring (10) 13.5–17.5 (–20.0) × 5.0–6.5 (7.0) μm with Q = 2.4–3.1, and association with plants of *Betula* sp. [81]. In our phylogenetic analysis of the multi-locus and ITS datasets (Figure 1 and Figure 2), *L. parascabrum* formed independent clades within *Leccinum*. It might represent a distinct section or subsection. However, formal change of the infrageneric division of this clade should await more molecular and morphological data from additional taxa. Species to which it is phylogenetically related remain as yet unknown.

**Figure 6:**
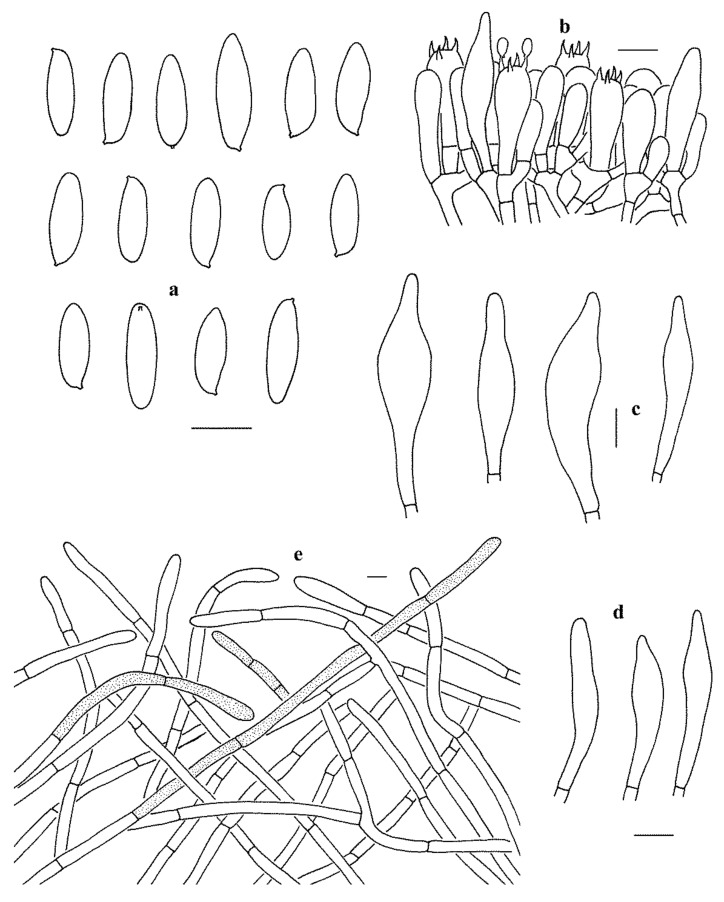
Microscopic features of *Leccinum pseudoborneense* (KUN-HKAS110156, holotype). (**a**) Basidiospores. (**b**) Basidia and pleurocystidia. (**c**) Pleurocystidia. (**d**) Cheilocystidia. (**e**) Pileipellis. Bars = 10 µm. Drawings by Y.-C. Li.

***Leccinum pseudoborneense*** X. Meng & Yan C. Li & Zhu L. Yang, sp. nov., (Figure 3d–f and Figure 6).

MycoBank: MB 838915.

*Diagnosis*: This species differs from other species in *Leccinum* in its nearly glabrous and pale brown to dark brown pileus, white context in pileus lacking color change when injured, white context in stipe staining blue when bruised, initially white and then brown hymenophore not changing color when injured, white stipe covered with ochraceous to dark brown squamules, and trichodermal pileipellis composed of 3–6 μm wide interwoven hyphae.

*Holotype*: CHINA. Yunnan Province: Xishuangbanna, Menghai County, Bada Town, E 100°12′, N 21°83′, alt. 1900 m, associated with *Castanopsis calathiformis*, *Castanopsis indica* and *Lithocarpus truncatus*, 22 June 2020, G.S. Wang 947 (KUN-HKAS110156, GenBank Acc. No.: MZ412902 for ITS, MW413908 for nrLSU, MW439268 for *tef1-α*, and MW439261 for *rpb2*)

*Etymology*: Latin “*pseudo*” = false, “*borneense*” = *L. borneense*, “*pseudoborneense*” is proposed because this species is similar to the species *L. borneense* originally described from Malaysia.

Basidiomata small to medium-sized. Pileus 4–10 cm diam, subhemispherical to convex or plano-convex; surface nearly glabrous, viscid when wet, pale brown (6D6–C5) to dark brown (6F6–E5); context 5–10 mm thick in the center, white (1A1), not changing color when bruised; Hymenophore adnate to depressed around apex of stipe; white (1A1) to pallid when young and becoming brown (6B5) when mature, not changing color when injured. Tubes 4–10 mm long, creamy white (1A1) when young, and becoming brownish yellow (5C7–8) when mature, not changing color when bruised; pores fine, no more than 1 mm wide. Stipe 10–15 × 2.1–2.9 cm, clavate, always swollen downwards; surface white (1A1), covered with ochraceous (2B3–5) to dark brown (6E7) squamules, staining asymmetric blue (23E7) when injured; context white (1A1), staining blue (23E7) when injured; basal mycelium white (1A1).

Basidiospores (100/5/5) (11–) 12–19 (–20) × 4–5 (–6) µm, Q = (2.75–) 3–3.58 (–3.6), Q_m_ = 3.31 ± 0.16, subfusiform to ellipsoid, slightly thick-walled (up to 0.5 μm thick), yellowish brown to olive brown in KOH, yellow-brown to dark olive-brown in Melzer’s Reagent. Basidia 18–30 × 8–9 µm, clavate, 4-spored, hyaline to yellowish in KOH, yellowish to brownish yellow in Melzer’s Reagent. Hymenophoral trama boletoid, hyphae cylindrical, 3–6 µm wide, hyaline to yellowish in KOH, yellowish to brownish yellow in Melzer’s Reagent. Cheilo- and pleurocystidia 28–40 × 4–10 µm, abundant, subfusiform to fusiform, thin-walled, yellowish to brownish yellow in KOH, brownish to yellow-brown in Melzer’s Reagent. Pileipellis a trichoderm, composed of more or less vertically arranged 5–12 µm wide filamentous hyphae, yellowish brown to brownish in KOH, brown to dark brown in Melzer’s Reagent. Pileal trama made up of 6–12 µm wide filamentous hyphae, thin-walled, hyaline to yellowish in KOH, yellowish to yellow in Melzer’s Reagent. Clamp connections absent in all tissues.

*Habitat and Distribution*: Scattered in tropical forests dominated by plants of the families Fagaceae (*Castanopsis calathiformis*, Ca. *orthacantha*, Ca. *indica*, *Lithocarpus truncatus*, *Li. mairei* and *Quercus griffithii*); on acidic, loamy or mossy, humid soils; moderately common in southwestern China (Yunnan Province).

*Additional specimens examined*: CHINA. Yunnan Province: Xishuangbanna, Menghai County, Bada Township, E 100°13′, N 21°84′, alt. 1900 m, associated with *Castanopsis calathiformis*, Ca. *indica* and *Lithocarpus truncatus*, 22 June 2020, G.S. Wang 960 (KUN-HKAS110157, GenBank Acc. No.: MZ412903 for ITS, MW413909 for nrLSU, MW439269 for *tef1-α*, and MW439262 for *rpb2*), the same location, 22 June 2020, G.S. Wang 965 (KUN-HKAS110158, GenBank Acc. No.: MZ412904 for ITS, MW413910 for nrLSU, MW439270 for *tef1-α*, and MW439263 for *rpb2*); Nanjian County, Gonglang Town, Huangcaoping, E 100°30′, N 24°54′, alt. 1200 m, associated with *Castanopsis orthacantha*, *Lithocarpus mairei* and *Quercus griffithii*, 30 June 2015, K. Zhao 773 (KUN-HKAS92401, GenBank Acc. No.: MZ536632 for nrLSU, MZ543307 for *tef1-α*, and MZ543309 for *rpb2*); Jinghong County, Dadugang Town, E 100°25′, N 21°26′, alt. 600 m, associated with *Castanopsis indica* and *Lithocarpus truncatus*, 30 June 2014, K. Zhao 476 (KUN-HKAS89139, GenBank Acc. No.: MZ536631 for nrLSU, MZ543306 for *tef1-α*, and MZ543308 for *rpb2*).

*Commentary: Leccinum pseudoborneense* is characterized by the nearly glabrous and pale brown to dark brown pileus, the white context in pileus not changing color when injured, the white context in stipe staining blue when bruised, the initially white and then brown hymenophore not changing color when injured, the white stipe covered with ochraceous to dark brown squamules, and the trichodermal pileipellis composed of 3–6 μm wide interwoven hyphae. *Leccinum pseudoborneense* is similar to *L. borneense* (Corner) E. Horak, originally described from Malaysia, in that they share a brown pileus, bluish color change of the context in stipe when bruised, and similar size of basidiospores. However, *L. borneense* differs from *L*. *pseudoborneense* in its yellow to olive yellow hymenophore staining blue when hurt, pale yellow to yellow pileal context staining blue when hurt, and deep yellow context in stipe staining blue but sometimes with reddish tint at base when injured [6,84]. *Leccinum pseudoborneense* is phylogenetically close to *L. album* in our phylogenetica analyses (Figure 1 and Figure 2). However, *L. album* has a white basidioma, white hymenophore staining indistinctly greenish blue when hurt, white context in pileus not changing color when injured, white context in stipe unchanging or only staining distinctly greenish blue at base when injured, and relatively broad basidiospores measuring 15–19 × 5–7 µm.

## 4. Discussion

The genus *Leccinum* was defined and recognized variously by different mycologists. In an early molecular study, *Leccinum* was shown to be polyphyletic and proposed to be restricted to the sections *Leccinum* and *Scabra* by Binder and Besl [4]. Subsequently, Bresinsky and Besl [32] erected a genus *Leccinellum* Bresinsky & Manfr. Binder, to accommodate *L.* section *Luteoscabra*, including species with yellow hymenophores and/or context. In this study, the phylogenetic inferences based on the multi-locus dataset of nrLSU, *tef1-α* and *rpb2* largely coincide with those of Binder and Besl [4], Bresinsky and Besl [32] and den Bakker et al. [33]. Thus, we adopt the treatment of Bakker et al. [33] and treat *Leccinum* in a strict circumscription, which only includes species of *L.* sect. *Leccinum* (Singer’s infrageneric classification with *L.* sect. *Scabra* merged to this section). Species in *Leccinum* are characterized by the white context lacking color changes or staining blue, gray or reddish tints when injured and the cutis-like pileipellis composed of interwoven filamentous hyphae

Eleven *Leccinum* species with specimen citations have been reported from China before this study, of which five species (*L. ambiguum*, *L. atrostipitatum*, *L. olivaceopallidum*, *L. potteri* and *L. subgranulosum*) were originally described from North America, five species (*L. aurantiacum*, *L. holopus*, *L. roseofractum*, *L. scabrum* and *L. versipelle*) were originally described from Europe, and only one taxon (*L. subleucophaeum* var. *minimum*) was originally described from China. Our molecular phylogenetic analyses along with morphological studies identified the existence of *L. quercinum*, *L. scabrum*, *L. subleucophaeum* var. *minimum* and *L. versipelle* in China. The distribution of other reported species have not yet been found, based on morphological and/or molecular data. In addition, three species new to science (*L. album*, *L. parascabrum* and *L. pseudoborneense*) and two species new to China (*L. melaneum* and *L. schistophilum*) were revealed in our study, based on molecular and morphology evidence. In conclusion, there are nine species of *Leccinum* in China.

Most species of *Leccinum* exhibit strong mycorrhizal host specificity. The host specificity along with climate type and edaphic factors appear to be important factors determining the distribution of different species. In China, *L.* *melaneum*, *L. scabrum*, *L. schistophilum* and *L. versipelle* are found in temperate forests and associated with plants of *Betula platyphylla* on acidic soils. *Leccinum album*, *L. parascabrum, L. pseudoborneense* and *L. subleucophaeum* var. *minimum* are found in tropical forests and associated with plants of Fagaceae (*Castanopsis calathiformis*, *Ca. hystrix*, *Ca. indica*, *Ca. orthacantha*, *Cyclobalanopsis* *glauca*, *Lithocarpus mairei*, *Li. truncatus* and *Quercus griffithii*) and/or Pinaceae (*Pinus kwangtudgensis* and *P. yunnanensis*) on acidic soils. It is noteworthy that *L. parascabrum* can be found in acidic or slightly alkaline habitats. *Leccinum versipelle* is found in subtropical forests and is associated with plants of *Populus yunnanensis* on acidic soils. The combination of the color of basidioma, the morphology of pileal surface, the size of basidiospores, the morphology of stipe, the color changes when injured, the climate type, the edaphic factors and the host preferences is very important in distinguishing species in this genus.

## Figures and Tables

**Table 1:** Information on specimens used in multi-locus phylogenetic analyses and their GenBank accession numbers. Sequences newly generated in this study are indicated in bold.

Species	Voucher	Locality	GenBank Number				Reference
			ITS	nrLSU	*tef1-α*	*rpb2*	
*Leccinellum corsicum*	Buf 4507	USA	-	KF030347	KF030435	-	[55]
*Le. crocipodium*	MICH:KUO-07050707	USA	-	MK601749	MK721103	MK766311	[5]
*Le.* aff. *griseum*	KPM-NC-0017381	Japan	-	JN378508	JN378449	-	[73]
*Le. lepidum*	K(M)-142974	Italy	-	MK601751	MK721105	MK766312	[5]
*Le. pseudoscabrum*	CFMR:DPL-11432	USA	-	MK601752	MK721106	MK766313	[5]
*Le. rugosiceps*	CFMR:BOS-866	USA	-	MK601770	MK721124	MK766329	[5]
*Leccinum album*	KUN-HKAS53417	China	MZ392872 MZ392873	MW413907 HQ326880	MW439267 HQ326861	MW439259 MW439260	**This study**
*L. aurantiacum*	L-0342207	France	-	MK601759	MK721113	MK766318	[5]
*L. cerinum*	MK11800	Finland	-	AF139692	-	-	[4]
*L. duriusculum*	KUN-HKAS101160	Uzbekistan	MZ485402	MZ675541	MZ707785	MZ707779	**This study**
*L. duriusculum*	GL4676	France	-	AF139699	-	-	[4]
*L. flavostipitatum*	MENMB10801	USA	-	MH620342	-	-	GenBank
*L. holopus*	MICH:KUO09150707	USA	-	MK601763	MK721117	MK766322	[5]
*L. holopus*	9109303	France	-	AF139700			[4]
*L. holopus*	KUN-HKAS111906	Austria	-	MW413906	MW439266	MW439258	**This study**
*L. manzanitae*	NY-14041	USA	-	MK601765	MK721119	MK766324	[5]
*L. melaneum*	KUN-HKAS57220	China	MZ485409	MZ675542	MZ707786	MZ707780	**This study**
*L. monticola*	NY-00815448	Costa Rica	-	MK601767	MK721121	MK766326	[5]
*L. monticola*	NY-760388	Costa Rica	-	MK601766	MK721120	MK766325	[5]
*L. palustre*	MK11107	Germany	-	AF139701	-	-	[4]
“*L. palustre*”	hdb030	Netherlands	AF454586	-	-	-	[57]
*L. parascabrum*	KUN-HKAS99903	China	MZ392874	MW413911	MW439271	MW439264	**This study**
*L. parascabrum*	KUN-HKAS59447	China	MZ392875	MW413912	MW439272	MW439265	**This study**
*L. pseudoborneense*	KUN-HKAS110156	China	MZ412902	MW413908	MW439268	MW439261	**This study**
*L. pseudoborneense*	KUN-HKAS110157	China	MZ412903	MW413909	MW439269	MW439262	**This study**
*L. pseudoborneense*	KUN-HKAS110158	China	MZ412904	MW413910	MW439270	MW439263	**This study**
*L. pseudoborneense*	KUN-HKAS89139	China	-	MZ536631	MZ543306	MZ543308	**This study**
*L. pseudoborneense*	KUN-HKAS92401	China	-	MZ536632	MZ543307	MZ543309	**This study**
*L. quercinum*	KUN-HKAS63502	China	-	KF112724	KF112250	KF112724	[65]
*L. quercinum*	Lq1	Germany	-	DQ534612	-	-	[74]
“*L. scabrum*”	KUN-HKAS56371	China	-	KT990587	KT990782	KT990423	[11]
*L. scabrum*	KUN-HKAS57266	China	-	KF112442	KF112248	KF112722	[65]
*L. scabrum*	KUN-HKAS98029	China	MZ485407	MZ675543	MZ707787	-	**This study**
*L. scabrum*	KPM-NC-0017840	UK	-	JN378515	JN378455	-	[73]
*L. schistophilum*	KUN-HKAS98024	China	MZ503508	MZ675544	MZ707788	-	**This study**
*L. schistophilum*	VDKO1128	Belgium	-	-	KT824055	KT824022	[75]
*L. subradicatum*	KPM-NC-24518	Japan	MT934814	MT812736	MT874822	-	[76]
*L.* sp.	KPM-NC-0017830	Japan	KC552009				[77]
*L. variicolor*	Lvar1	Germany	-	AF139706	-	-	[4]
*L. variicolor*	Hdb327	Canada	AY538843	-	-	-	[33]
*L. variicolor*	Watling6753	UK	AY853538	-	-	-	[78]
*L. versipelle*	KUN-HKAS76669	China	-	KF112443	KF112249	KF112723	[65]
*L. versipelle*	CFMR DLC2002-122	USA	-	MK601778	-	-	[5]
*L. versipelle*	-	Sweden	AF454573	-	-	-	[57]
*L. versipelle*	KUN-HKAS97997	China	MZ485404	MZ675545	MZ707789	MZ707781	**This study**
*L. versipelle*	KUN-HKAS99380	China	MZ485401	MZ675546	MZ707790	MZ707782	**This study**
*L. violaceotinctum*	CFMR:BZ-1676	Belize	-	MK601780	MK721133	MK766337	[5]
*L. violaceotinctum*	CFMR:BZ-3169	Belize	-	-	MK721134	MK766338	[5]
*Borofutus dhakanus*	KUN-HKAS73789	Bengal	-	JQ928616	JQ928576	JQ928597	[72]
*Chamonixia brevicolumna*	DBG:F023359	USA	-	MK601728	MK721082	MK766290	[5]
*Octaviania japonimontana*	KPM-NC-0017812	Japan	-	JN378486	JN378428	-	[73]
*O. tasmanica*	NY-02449788	USA	-	MK601798	MK721152	MK766355	[5]
*Rossbeevera griseobrunnea*	GDGM45913	China	-	MH537793	-	-	[79]
*R. eucyanea*	KPM-NC-0023895	Japan	-	KP222896	KP222915	-	[76]
*Rugiboletus andinus*	NY-00796145	USA	-	MK601758	MK721112	MK766317	[5]
*Ru. andinus*	NY-181460	USA	-	MK601757	MK721111	MK766316	[5]
*Spongiforma thailandica*	DED7873	Thailand	-	NG_042464	KF030436	MG212648	[71]
*Turmalinea mesomorpha*	KPM-NC-0017743	Japan	-	KC552050	-	-	[77]
*T. yuwanensis*	KPM-NC0023377	Japan	-	KJ001098	KJ001083	-	[77]
*Tylocinum griseolum*	KUN-HKAS52612	China	-	KT990631	KT990825	-	[11]

## Data Availability

Publicly available datasets were analyzed in this study. This data can be found here: https://www.ncbi.nlm.nih.gov/; http://www.mycobank.org/; https://www.treebase.org/treebase-web/home.html, accessed on 26 August 2021.

## Supplementary Materials

The following are available online at https://www.mdpi.com/article/10.3390/jof7090732/s1, Alignment S1: alignment of multi-locus dataset; Alignment S2: alignment of ITS dataset.

## References

[1] Smith A.H., Thiers H.D., Watling R. (1968). Notes on species of *Leccinum*. I. Additions to section *Leccinum*. Lloydia.

[2] Engel H., Krieglsteiner G.J. (1978). Rauhstielröhrlinge–die Gattung Leccinum in Europa.

[3] Singer R. (1986). The Agaricales in Modern Taxonomy.

[4] Binder M., Besl H., Associazone Micologica Bresadola (2000). 28S rDNA sequence data and chemotaxonomical analyses on the generic concept of *Leccinum* (Boletales). Micologia.

[5] Kuo M., Ortiz-Santana B. (2020). Revision of leccinoid fungi, with emphasis on North American taxa, based on molecular and morphological data. Mycologia.

[6] Corner E.J.H. (1972). Boletus in Malaysia.

[7] Nagasawa E. (1997). A preliminary checklist of the Japanese agaricales, 1: The boletineae. Rep. Tottori. Mycol. Inst..

[8] Takahashi H. (2007). Five new species of the Boletaceae from Japan. Mycoscience.

[9] Terashima Y., Takahashi H., Taneyama Y. (2016). The Fungal Flora in Southwestern Japan: Agarics and Boletes.

[10] Katumoto K. (2010). List of Fungi Recorded in Japan.

[11] Wu G., Li Y.C., Zhu X.T., Zhao K., Han L.H., Cui Y.Y., Li F., Xu J., Yang Z.L. (2016). One hundred noteworthy boletes from China. Fungal Divers..

[12] Li T.H., Song B. Chinese boletes: A comparison of boreal and tropical elements. Proceedings of the Tropical Mycology 2000, the Millennium Meeting on Tropical Mycology (Main Meeting 2000), British Mycological Society & Liverpool John Moores University.

[13] Gray S.F., Gray J.E., Shury J. (1821). A Natural Arrangement of British Plants.

[14] Den Bakker H.C., Noordeloos M.E. (2005). A revision of European species of *Leccinum* Gray and notes on extralimital species. Persoonia.

[15] Smith A.H., Thiers H.D., Watling R. (1966). A preliminary account of the North American species of *Leccinum*, Section *Leccinum*. Mich. Bot..

[16] Smith A.H., Thiers H.D., Watling R. (1967). A Preliminary account of the North American species of *Leccinum*, Sections *Luteoscabra* and *Scabra*. Mich. Bot..

[17] Smith A.H., Thiers H.D. (1971). The Boletes of Michigan.

[18] Thiers H.D. (1971). California *Boletes*. IV. The Genus *Leccinum*. Mycologia.

[19] Both E. (1993). The Boletes of North America, a Compendium.

[20] Ortiz-Santana B., Halling R.E. (2009). A new species of *Leccinum* (Basidiomycota, Boletales) from Belize. Brittonia.

[21] Halling R.E., Mueller G.M. (2003). *Leccinum* (Boletaceae) in Costa Rica. Mycologia.

[22] Halling R.E., Mueller G.M. (2005). Common Mushrooms of the Talamanca Mountains, Costa Rica.

[23] Halling R.E. (1999). New *Leccinum* from Costa Rica. Kew Bull..

[24] Halling R.E. (1989). A synopsis of Colombian boletes. Mycotaxon.

[25] Assyov B., Denchev C.M. (2004). Preliminary checklist of Boletales s. str. in Bulgaria. Mycol. Balc..

[26] Segedin B.P., Pennycook S.R. (2001). A nomenclatural checklist of agarics, boletes, and related secotioid and gasteromycetous fungi recorded from New Zealand. N. Z. J. Bot..

[27] Watling R. (2001). Australian boletes: Their diversity and possible origins. Aus. Syst. Bot..

[28] Segedin P.B. (1987). An annotated checklist of Agarics and Boleti recorded from New Zealand. N. Z. J. Bot..

[29] Mcnabb R.F.R. (1968). The Boletaceae of New Zealand. N. Z. J. Bot..

[30] Peck C.H. (1888). Report of the botanist. Ann. Rep. N. Y. St. Mus. Nat. Hist..

[31] Šutara J. (1989). The delimitation of the genus *Leccinum*. Czech. Mycol..

[32] Bresinsky A., Besl H. (2003). Beiträge zu einer Mykoflora Deutschlands: Schlüssel zur Gattungsbestimmung der Blätter-, Leisten- und Röhrenpilze mit Literaturhinweisen zur Artbestimmung. Regensb. Mykol. Schr..

[33] Den Bakker H.C., Zuccarello G.C., Kuyper T.W., Noordeloos M.E. (2004). Evolution and host specificity in the ectomycorrhizal genus *Leccinum*. New Phytol..

[34] Zang M. (1986). Notes on the boletales from Eastern Himalayas and adjacent areas of China. Acta Bot. Yunnanica.

[35] Wu G., Zhao K., Li Y.C., Zeng N.K., Feng B., Halling R.E., Yang Z.L. (2016). Four new genera of the fungal family Boletaceae. Fungal Divers..

[36] Wu K., Wu G., Yang Z.L. (2020). A taxonomic revision of *Leccinum rubrum* in subalpine coniferous forests, Southwestern China. Acta Edulis Fungi.

[37] Halling R.E., Fechner N., Nuhn M., Osmundson T., Soytong K., Arora D., Binder M., Hibbett D. (2015). Evolutionary relationships of *Heimioporus* and *Boletellus* (Boletales) with an emphasis on Australian taxa including new species and new combinations in *Aureoboletus*, *Hemileccinum*, and *Xerocomus*. Aust. Syst. Bot..

[38] Chiu W.F. (1948). The boletes of Yunnan. Mycologia.

[39] Mao X.L., Jiang C.P., Ouzhu C.W. (1993). Economic Macrofungi of Tibet.

[40] Mao X.L. (1998). The Economic Fungi in China.

[41] Mao X.L. (2000). The Macrofungi of China.

[42] Wu X.L., Zang M., Xia T.Y. (1997). Coloured Illustrations of the Ganodermataceae and other Fungi.

[43] Ying J.Z., Wen H.A., Zong Y.C. (1994). The Economic Macromycetes from Western Sichuan.

[44] Bi Z.S., Zheng G.Y., Li T.H., Wang Y.Z. (1990). Macrofungus Flora of the Mountainous District of North. Guangdong.

[45] Yuan M.S., Sun P.Q. (1995). Sichuan Mushrooms.

[46] Bi Z.S., Li T.H., Zhang W.M., Song B. (1997). A Preliminary Agaric Flora of Hainan Province.

[47] Yeh K.W., Chen Z.C. (1980). The boletes of Taiwan, I. Taiwania.

[48] Bi Z.S., Li T.H., Zheng G.Y., Li C. (1984). Basidiomycetes from Dinghu Mountain of China. III. Some species of Boletaceae (2). Acta Myco. Sinica.

[49] Song B., Li T.H., Wu X.L., Shen Y.H. (2004). A preliminary review on Boletales resources in Yunnan, Guizhou and Guangxi, China. Guizhou Sci..

[50] Fu S.Z., Wang Q.B., Yao Y.J. (2006). An annotated checklist of *Leccinum* in china. Mycotaxon.

[51] Li T.H., Song B. (2003). Bolete species known from China. Guizhou Sci..

[52] Li Y.C., Feng B., Yang Z.L. (2011). *Zangia*, a new genus of Boletaceae supported by molecular and morphological evidence. Fungal Divers..

[53] Halling R.E., Nuhn M.E., Fechner N., Osmundson T., Soytong K., Arora D., Hibbet D.S., Binder M. (2012). *Sutorius*: A new genus for *Boletus eximius* (Boletaceae). Mycologia.

[54] Halling R.E., Nuhn M.E., Osmundson T., Fechner N., Trappe J.M., Soytong K., Arora D., Hibbett D.S., Binder M. (2012). Affinities of the *Boletus chromapes* group to *Royoungia* and the description of two new genera, *Harrya* and *Australopilus*. Austral. Syst. Bot..

[55] Nuhn M.E., Binder M., Taylor A.F.S., Halling R.E., Hibbett D.S. (2013). Phylogenetic overview of the boletineae. Fungal Biol..

[56] Wu F., Zhou L.W., Yang Z.L., Bau T., Li T.H., Dai Y.C. (2019). Resource diversity of Chinese macrofungi: Edible, medicinal and poisonous species. Fungal Divers..

[57] Den Bakker H.C., Gravendeel B., Kuyper T.W. (2004). An ITS Phylogeny of *Leccinum* and an Analysis of the Evolution of Minisatellite-like Sequences within ITS1. Mycologia.

[58] Kornerup A., Wanscher J.H. (1983). Methuen Handbook of Colour.

[59] Zhou M., Dai Y.C., Vlasák J., Yuan Y. (2021). Molecular Phylogeny and Global Diversity of the Genus *Haploporus* (Polyporales, Basidiomycota). J. Fungi.

[60] Doyle J.J., Doyle J.L. (1987). A rapid DNA isolation procedure for small quantities of fresh leaf tissue. Phytochem. Bullet..

[61] Vilgalys R., Hester M. (1990). Rapid genetic identification and mapping of enzymatically amplified ribosomal DNA from several *Cryptococcus* species. J. Bact..

[62] Rehner S.A., Buckley E. (2005). A *Beauveria* phylogeny inferred from nuclear ITS and *EF1-α* sequences: Evidence for cryptic diversification and links to *Cordyceps teleomorphs*. Mycologia.

[63] Matheny P.B. (2005). Improving phylogenetic inference of mushrooms with *rpb1* and *rpb2* nucleotide sequences (*Inocybe*, Agaricales). Mol. Phylogenet. Evol..

[64] White T.J., Bruns T., Lee S., Taylor J., Innis M.A., Gelfand D.H., Sninsky J.J., White T.J. (1990). Amplification and direct sequencing of fungal ribosomal RNA genes for phylogenetics. PCR Protocols. A Guide to Methods and Applications.

[65] Wu G., Feng B., Xu J., Zhu X.T., Li Y.C., Zeng N.K., Hosen M.I., Yang Z.L. (2014). Molecular phylogenetic analyses redefine seven major clades and reveal 22 new generic clades in the fungal family Boletaceae. Fungal Divers..

[66] Katoh K., Standley D.M. (2013). MAFFT multiple sequence alignment software version 7: Improvements in performance and usability. Mol. Biol. Evol..

[67] Hall T.A. (1999). BioEdit: A user-friendly biological sequence alignment editor and analyses program for Windows 95/98/NT. Nucleic Acids Symp. Ser..

[68] Stamatakis A., Hoover P., Rougemont J. (2008). A rapid bootstrap algorithm for the RAxML web-servers. Syst. Biol..

[69] Posada D., Buckley T.R. (2004). Model selection and model averaging in phylogenetics: Advantages of the AIC and Bayesian approaches over likelihood ratio tests. Syst. Biol..

[70] Huelsenbeck J.P., Ronquist F., Nielsen R. (2005). Bayesian analysis of molecular evolution using MrBayes. Statistical Methods in Molecular Evolution.

[71] Desjardin D.E., Binder M., Roekring S., Flegel T. (2009). *Spongiforma*, a new genus of gasteroid boletes from Thailand. Fungal Divers..

[72] Hosen M.I., Feng B., Wu G., Zhu X.T., Li Y.C., Yang Z.L. (2013). *Borofutus*, a new genus of Boletaceae from tropical Asia: Phylogeny, morphology and taxonomy. Fungal Divers..

[73] Orihara T., Smith M.E., Shimomura N., Iwase K., Maekawa N. (2012). Diversity and systematics of the sequestrate genus *Octaviania* in Japan: Two new subgenera and eleven new species. Persoonia.

[74] Binder M., Hibbett D. (2006). Molecular systematics and biological diversification of Boletales. Mycologia.

[75] Raspé O., Vadthanarat S., Kesel A.D., Degreef J., Hyde K.D., Lumyong S. (2016). *Pulveroboletus fragrans*, a new Boletaceae species from Northern Thailand, with a remarkable aromatic odor. Mycol. Progr..

[76] Orihara T., Healy R., Corrales A., Smith M.E. (2021). Multi-locus phylogenies reveal three new truffle-like taxa and the traces of interspecific hybridization in *Octaviania* (Boletales). IMA Fungus.

[77] Orihara T., Lebel T., Ge Z.W., Smith M.E., Maekawa N. (2016). Evolutionary history of the sequestrate genus *Rossbeevera* (Boletaceae) reveals a new genus *Turmalinea* and highlights the utility of ITS minisatellite-like insertions for molecular identification. Persoonia.

[78] Den Bakker H.C., Zuccarello G.C., Kuyper T.W., Noordeloos M.E. (2007). Phylogeographic patterns in *Leccinum* sect. Scabra and the status of the arctic-alpine species L. rotundifoliae. Mycol. Res..

[79] Hosen M.I., Zhong X.J., Gates G., Orihara T., Li T.H. (2019). Type studies of *Rossbeevera bispora*, and a new species of *Rossbeevera* from south China. MycoKeys.

[80] Watling R. (1970). British Fungus Flora—Agarics and Boleti. I. Boletaceae, Gomphidiaceae, Paxillaceae.

[81] Rostkovius F.W.T., Sturm J. (1832). Die Pilze Deutschlands. Deutschlands Flora in Abbildungen nach der Natur mit Beschreibungen.

[82] Watling R. (1960). British records. Trans. Br. mycol. Soc..

[83] Noordeloos M.E., Kuyper T.W., Somhorst I., Vellinga E.C. (2018). Flora Agaricina Neerlandica.

[84] Horak E. (2011). Revision of Malaysian species of Boletales s.l. (Basidiomycota) described by E.J.H. Corner (1972, 1974). Malay. For. Rec..

